# Prevalence of ADHD Among Saudi Children and Adolescents

**DOI:** 10.3390/ijerph23040436

**Published:** 2026-03-30

**Authors:** Faisal O. AlQurashi, Renad A. Alrasan, Reem N. Mohamed, Nora M. Alzahrani, Alea A. Mohammedhussain, Nersyan N. Sharbini, Bayan M. Almanasif

**Affiliations:** 1 Department of Pediatrics, College of Medicine, King Fahad Hospital of the University, Imam Abdulrahman Bin Faisal University, Dammam 34224, Saudi Arabia; 2 College of Medicine, Imam Abdulrahman Bin Faisal University, Dammam 34224, Saudi Arabia; 3 Department of Pediatrics, Dr. Sulaiman Al Habib Hospital, Al Khobar 34423, Saudi Arabia; 4 Nursing Department, King Fahad Hospital of the University, Imam Abdulrahman Bin Faisal University, Dammam 34224, Saudi Arabia

**Keywords:** attention-deficit/hyperactivity disorder (ADHD), neurodevelopmental disorders, behavioral disorders, ADHD prevalence, Saudi Arabia

## Abstract

**Highlights:**

**Public health relevance—How does this work relate to a public health issue?**

- Attention-deficit/hyperactivity disorder (ADHD) is a common neurodevelopmental disorder that affects children’s academic performance, behavior, and long-term psychosocial functioning.
- Regional epidemiological data on ADHD in Saudi Arabia are limited, hindering early identification, prevention, and targeted mental health planning.

**Public health significance—Why is this work of significance to public health?**

- This large cross-sectional study estimates ADHD prevalence (12.7%) among children and adolescents in the Eastern Province and identifies clinical, developmental, and familial risk factors.
- Findings demonstrate substantial functional impairment and high psychiatric comorbidity, highlighting ADHD as an important contributor to childhood mental health burden.

**Public health implications—What are the key implications or messages for practitioners, policy makers and/or researchers in public health?**

- Early screening and school-based detection programs, particularly for high-risk children (e.g., low birth weight or family psychiatric history), may improve timely diagnosis and intervention.
- Results support strengthening child mental health services, preventive strategies, and family-centered care to reduce long-term educational and social consequences.

**Abstract:**

Attention-deficit hyperactivity disorder (ADHD) is a neurodevelopmental disorder that begins in childhood and may persist into adulthood, characterized by inattention, impulsivity, and hyperactivity leading to functional impairment. The global prevalence in children and adolescents ranges from 5–7%, yet data from the Eastern Province of Saudi Arabia remain limited. This study determined the prevalence of hyperactive/impulsive and inattentive symptoms among children and adolescents aged 4–18 years and identified associated factors. A cross-sectional study was conducted across major cities using validated Arabic versions of the SNAP-IV and NICHQ Vanderbilt Assessment Scale distributed online. The survey assessed ADHD symptoms, oppositional defiant disorder, conduct disorder, anxiety/depression, functional impairment, and prenatal and perinatal risk factors, with DSM-based scoring and multivariable logistic regression to identify predictors. Among 920 participants (mean age 10.7 years; 52.9% boys), 12.7% met criteria for ADHD. The inattentive subtype was most common, followed by combined and hyperactive/impulsive types. Affected children and adolescents showed academic and interpersonal difficulties. Significant predictors included younger age, birth weight <1.5 kg, psychiatric history, previous ADHD diagnosis, and family history of psychiatric or neurological disorders. ADHD symptoms were relatively common and associated with functional impairment and identifiable developmental and familial risk factors.

## 1. Introduction

The mental health of children and adolescents is a critical global issue that significantly affects their development, academic achievement, social relationships, and overall well-being. The early onset of mental health conditions can lead to long-term consequences if left unaddressed, including poor educational outcomes, substance abuse, and increased risk of suicide. Globally, an estimated 10–20% of children and adolescents experience mental health disorders; however, many cases in low- and middle-income countries remain undiagnosed and untreated. Therefore, promoting mental well-being from an early age is essential for building a healthier society [1].

Among childhood mental health disorders, attention-deficit hyperactivity disorder (ADHD) is one of the most common neurodevelopmental conditions. ADHD typically manifests during childhood and can persist into adulthood. It is characterized by inattention, impulsivity, and hyperactivity, resulting in functional impairment in social, occupational, or academic activities. ADHD is clinically diagnosed using the DSM-5 diagnostic criteria, and evaluation should include an assessment of the differential diagnosis of ADHD. Given the substantial number of affected children and adolescents, ADHD represents a significant public health concern. The estimated global prevalence of ADHD in children and adolescents ranges from 5% to 7%, although reported rates vary owing to differences in diagnostic criteria, assessment methods, and cultural perceptions of behavior [2].

ADHD prevalence estimates vary owing to differences in study methods, variations in age groups, and evolving diagnostic criteria. For example, a recent meta-analysis reported a global ADHD prevalence of approximately 7.2% in children, with some community-based studies reporting rates ranging from 8.7% to 15.5% [3].

More recently, a 2023 study published in the Journal of Affective Disorders provided a comprehensive synthesis of the global ADHD prevalence among children and adolescents through an umbrella review of multiple meta-analyses. This review integrated data from 13 systematic reviews and meta-analyses, encompassing 588 primary studies and more than 478,000 diagnosed cases of ADHD among over 3.2 million participants worldwide. These studies spanned diverse regions, including Africa, Asia, Europe, and the Americas. The review estimated an overall ADHD prevalence of approximately 8% in youths, with relatively consistent rates globally, except for notably higher prevalence observed in Black, White, and Asian American populations, as well as in India and Middle Eastern countries [4].

In the United States, a cross-sectional study published in 2018 estimated the national prevalence of parent-reported ADHD diagnoses and treatment among children aged 2–17 years using data from the 2016 National Survey of Children’s Health (NSCH). The study reported a weighted prevalence estimate of 9.4%, corresponding to approximately 6.1 million children who had received an ADHD diagnosis [5].

Within Saudi Arabia, several studies have examined ADHD prevalence in specific regions. In one cross-sectional study published in 2018, the aim was to determine the prevalence of ADHD and its subtypes, and to explore associated psychiatric, academic, and behavioral comorbidities in public primary school students in Jeddah, Saudi Arabia. Teachers assessed students using the Vanderbilt ADHD Diagnostic Teacher Rating Scale (VADTRS). Among 929 students aged 6–12 years, the overall prevalence of ADHD was 5%. The most prevalent subtype of ADHD was the combined type (2.7%), followed by the hyperactive (1.2%) and inattentive (1.1%) subtypes [6].

Despite these findings, there remains a notable scarcity of research on ADHD symptoms among children and adolescents in the Eastern Province of Saudi Arabia, particularly when compared with other regions such as Riyadh and Jeddah. This gap highlights the urgent need for region-specific research to inform local healthcare policies and target interventions. Without reliable data from the Eastern Province, healthcare planners and educators lack sufficient evidence to allocate resources effectively, design culturally appropriate screening tools, or implement school-based support programs. Moreover, local data are essential for capturing the unique social, cultural, and environmental factors that may influence how ADHD symptoms manifest and are perceived.

In the Eastern Province, a cross-sectional study was performed among girls and boys aged 6–10 years, including a sample size of 1658 children, of whom 185 children were identified as having ADHD [7]. Outside the Eastern Province, another cross-sectional study among public primary school students in Jeddah reported an overall ADHD prevalence of 5%, with rates of 5.3% among girls and 4.7% among boys [6]. However, a study conducted in Al-Khobar City within the Eastern Province, among female primary school children, reported an overall ADHD prevalence of 3.5%, with 2.1% inattentive and 5.6% hyperactive impulsive subtypes [8].

This study is based on the hypothesis that the prevalence of ADHD symptoms among children and adolescents aged 4–18 years exceeds the prevalence of formally diagnosed ADHD cases reported by child psychiatry clinics. Furthermore, we hypothesize that several factors may influence the development and expression of ADHD symptoms, including sociodemographic characteristics, history of head trauma, exposure to psychological stressors, and maternal or familial history of ADHD or other psychiatric disorders.

Although ADHD and its associated developmental and familial correlates have been widely studied internationally, important epidemiologic gaps remain in underrepresented settings, including the Eastern Province of Saudi Arabia, where available data are limited and heterogeneous in age range, setting, and informant source. In this context, the value of the present study lies not in proposing entirely novel predictors, but in providing contemporary, region-specific evidence from a broad sample of children and adolescents aged 4–18 years across multiple cities in the Eastern Province using a validated Arabic Vanderbilt-based assessment framework. By examining probable ADHD prevalence together with subtype distribution, functional impairment, psychiatric comorbidity, and adjusted associations with selected developmental and familial factors, this study adds cross-cultural and public health context to the international ADHD literature and contributes data from a Middle Eastern population that remains comparatively underrepresented in global epidemiologic research. Accordingly, this study aimed to estimate the prevalence of probable ADHD symptoms in children and adolescents in the Eastern Province of Saudi Arabia and to evaluate their distribution, associated impairment, and independently associated factors.

## 2. Materials and Methods

### 2.1. Study Design

This cross-sectional study was conducted over a 12-month period from February 2024 to February 2025 to assess the prevalence of ADHD symptoms among children and adolescents in the Eastern Province of Saudi Arabia. This study was conducted across major urban centers in the Eastern Province, including Dammam, Khobar, Qatif, Jubail, and Al-Ahsa, in addition to several smaller municipalities within the region. It targeted children and adolescents aged 4–18 years who were enrolled in both public and private schools in the region. Individuals over the age of 18 years, those living outside the Eastern Province, and parents who did not complete the questionnaire were excluded from the study.

### 2.2. Survey Design and Participants

A proxy-reported questionnaire by parents/guardians was developed by the authors using the Google Forms web application. The questionnaire was based on the Arabic and English versions of the SNAP-IV 26-Item Teacher and Parent Rating Scale and the NICHQ Vanderbilt Assessment Scale. For the Arabic version, we utilized the officially translated and validated Vanderbilt Assessment Scale provided by the Saudi ADHD Society [9]. It was distributed to all eligible participants via social media platforms, such as WhatsApp and X (formerly Twitter). In addition, school principals were contacted formally to encourage parents and teachers to participate. The questionnaire was divided into several sections, beginning with an introduction that outlined the study’s objectives and required participants’ consent before proceeding to the next section.

Participants indicated their relationship with the child (father, mother, or other relative) and reported their age group (<20, 20–25, 26–30, 31–35, 36–40, and >40 years), as well as their educational attainment (illiterate, high school, diploma, bachelor’s degree, master’s degree, or PhD). They also provided the participants’ precise age (4–18 years) and sex. Respondents were asked whether the child had a prior psychiatric diagnosis, specifically ADHD, or another psychiatric disorder. If ADHD was indicated, additional information regarding medication status (current, never, previously on medication, or not applicable) was collected.

Two core symptom domains, including inattention (items 1–9) and hyperactivity/impulsivity (items 10–18), were assessed using a four-point frequency Likert scale: 0 = never, 1 = occasionally, 2 = often, and 3 = very often. Positive symptoms were defined as responses of 2 or 3. Consistent with the DSM-IV criteria, participants were classified as meeting symptom criteria if they endorsed six or more positive items in either domain.

Additional scales assessed oppositional defiant disorder (items 19–26), conduct disorder (items 27–40), and anxiety/depression symptoms (items 41–47). These domains used the same four-point Likert scale, and predetermined symptom thresholds for each domain were applied to screen for possible comorbidities.

Functional impact was evaluated using eight items (items 48–55) covering areas such as school performance, reading, writing, mathematics, relationships with parents, siblings, peers, and participation in organized activities. Response options were as follows: 1 = excellent, 2 = above average, 3 = average, 4 = somewhat problematic, and 5 = problematic. ADHD case identification required at least one score of 4 or 5, indicating impairment. In addition, a separate item (item 56) asked whether the child’s school had contacted the guardian regarding issues such as hyperactivity, inattention, distractibility, or impulsivity, with response options of “always,” “sometimes,” or “never”.

Section 9 of the questionnaire describes potential prenatal and perinatal risk factors. These included birth weight categorized as >4 kg, 2.5–4 kg, 1.5–2.5 kg, or <1.5 kg; mode of delivery, classified as normal vaginal delivery, elective cesarean section, emergency cesarean section, or unknown; and gestational age, classified as preterm (<37 weeks), term (≥37 weeks), or unknown. The section also recorded any history of meningitis, head trauma, or abnormal neuroimaging findings, as well as prenatal exposure to tobacco, alcohol, or recreational drugs (marked as yes, no, or unknown). In addition, it included information on family history of neurological, developmental, or psychiatric disorders (yes/no/unknown) and the child’s average daily screen time, grouped as <1, 1–2, 2–3, or >3 h.

The scoring and case definition of ADHD involved several components. Symptom scores were determined by summing the positive responses (rated 2 or 3) separately for inattention and hyperactivity/impulsivity, and the total symptom score was calculated across all 18 items. Functional impairment was assessed by requiring at least one performance item to score ≥ 4 to meet ADHD criteria. In addition, syndrome screening involved tallying positive counts in the domains of oppositional defiant disorder (ODD), conduct disorder, and anxiety/depression, which were interpreted according to established thresholds. A case was classified as probable ADHD if it met three criteria: six or more positive symptoms in either the inattention or hyperactivity/impulsivity domain; at least one indication of functional impairment (performance score ≥ 4); and evidence that symptoms affected multiple settings, such as school and home, corroborated by school contact reports.

The survey was administered online and required approximately 10–15 min to complete. Automated branching logic was used to guide participants through the relevant sections, and data integrity checks flagged incomplete or inconsistent records for review. Unique anonymous identifiers are used to ensure confidentiality.

Pilot testing was conducted with a small sample (*n* ≈ 20) to assess clarity and completion time, and refinements were made prior to full administration. The instrument’s content mirrored validated scales such as the Vanderbilt ADHD Diagnostic Rating Scale and SNAP-IV in structure, although it was tailored to the local context and translated where necessary. Participants were encouraged to respond honestly, and reminders were included to maximize data quality.

### 2.3. Statistical Analysis

IBM SPSS Statistics for Windows, version 26.0 (IBM Corp., Armonk, NY, USA) was used for data analysis. Continuous variables were summarized using means and standard deviations, and categorical variables were presented as frequencies and percentages. Bivariate analyses were conducted as exploratory, unadjusted comparisons using the chi-square test or Fisher’s exact test, as appropriate, and independent *t*-tests for continuous variables. Because several of the examined variables were clinically related, formal Bonferroni correction was not applied, as this approach may be overly conservative in this context. Therefore, *p*-values from bivariate analyses were interpreted cautiously and in conjunction with effect estimates and the results of the multivariable logistic regression model. Independent-samples *t*-tests were used for continuous comparisons. Given the large sample size, these parametric comparisons were considered robust to minor deviations from normality. Multivariable logistic regression analysis was then performed to identify independent factors associated with ADHD, and both unadjusted and adjusted odds ratios with 95% confidence intervals were reported.

## 3. Results

This study included 920 children and adolescents, with a mean age of 10.7 years. Boys represented more than half of the sample (52.9%), and most of the responding relatives were mothers (56.1%), followed by fathers (16.5%) and other relatives (27.4%). The majority of relatives were older than 30 years, with nearly one-third of them above 40 years of age. In terms of education, more than half of the caregivers held a bachelor’s degree (55.5%), whereas only a small proportion were illiterate (0.8%). Regarding psychiatric conditions, 21.0% of the participants had a previous ADHD diagnosis, and 4.8% had other psychiatric disorders, while almost three-quarters (74.2%) had no reported diagnosis. The term “other psychiatric disorder” refers generally to any possible diagnosed co-existing psychiatric disorder. Among the children and adolescents with ADHD, only a minority were currently receiving medication (5.7%), and (3.5%) of participants were previously on ADHD medication. Based on the NICHQ Vanderbilt Assessment Scale, 117 participants (12.7%) were diagnosed with ADHD. When comparing children and adolescents classified as having ADHD with those without ADHD symptoms (Non-ADHD group), no significant differences were observed in terms of child age, sex, caregiver relationship, or caregiver’s level of education. However, the presence of previous psychiatric comorbidities strongly distinguished the groups; nearly half of the participants with ADHD had been previously diagnosed with ADHD, compared to only 17.3% in the non-ADHD group (*p* < 0.001; Table 1).

Birth-related factors also appear to be relevant. Children and adolescents with ADHD were more likely to have been born via emergency cesarean section (19.7% of participants with ADHD vs. 10.5% of participants without ADHD *p* = 0.033), although gestational age at birth and birth weight were not significantly associated with ADHD status. Several clinical and family history variables were strongly associated with ADHD. A history of meningitis (*p* = 0.006), head trauma (*p* = 0.0015), and abnormal brain imaging findings (*p* < 0.001) were significantly more common in participants with ADHD. In addition, almost half (41.0%) of the ADHD cases reported a positive family history of psychiatric or neurological disorders, compared to only 16.9% in the non-ADHD group (*p* < 0.001). Screen time did not differ significantly between the groups, although children and adolescents with ADHD tended to spend longer periods on digital devices. Maternal exposure to tobacco, alcohol, and medications during pregnancy was slightly higher in the ADHD group; however, this difference was not statistically significant (Table 2).

**Figure 1:**
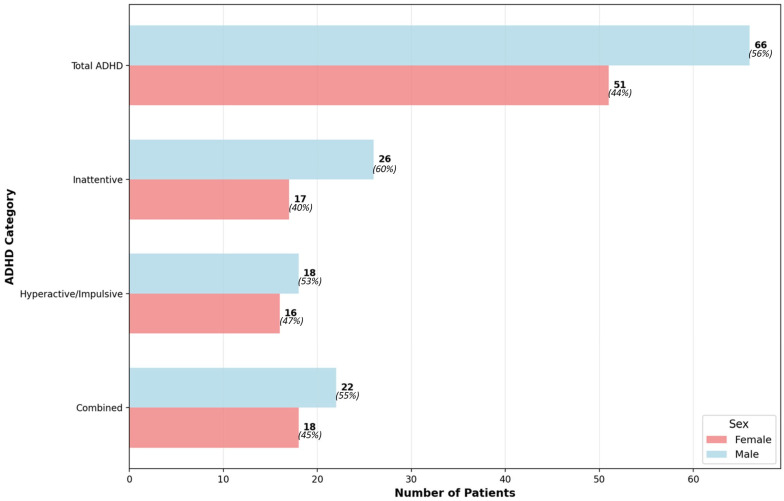
Distribution of ADHD and its subtypes based on the NICHQ Vanderbilt Assessment Scale regarding the sex of the participants.

As shown in Figure 1, ADHD was more frequently observed among boys than girls (66 vs. 51 participants). Across subtypes, the inattentive presentation was the most common, followed by the combined and hyperactive/impulsive types. The sex distribution revealed that boys consistently outnumbered girls in each ADHD subtype.

**Figure 2:**
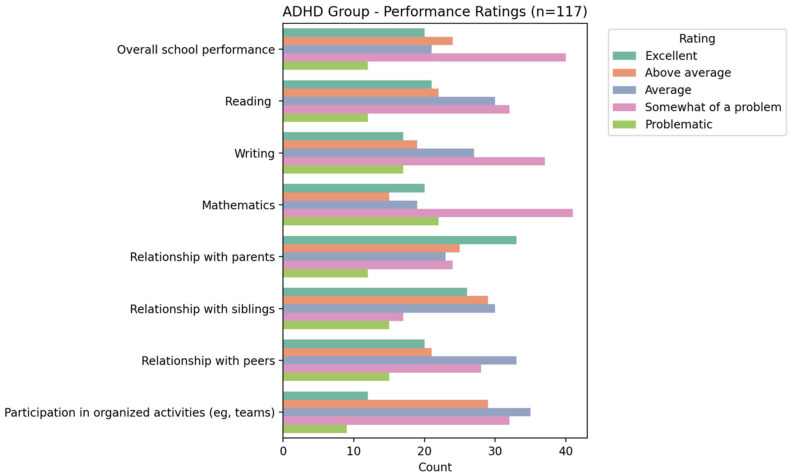
Distribution of performance rating among children and adolescents with ADHD.

**Figure 3:**
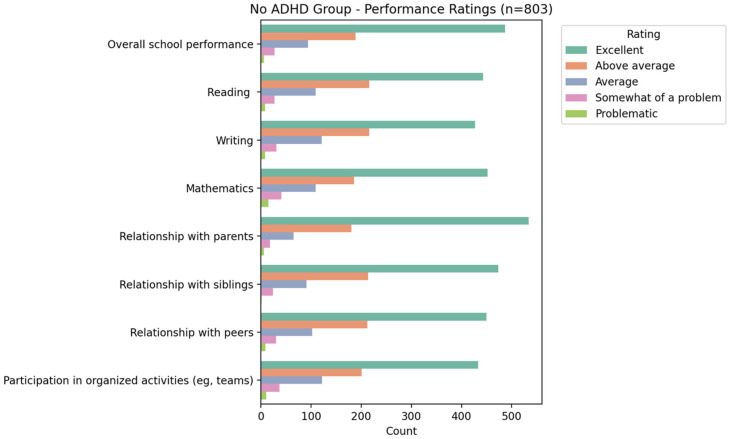
Distribution of performance rating among non-ADHD children and adolescents.

Based on the NICHQ Vanderbilt Assessment Scale, participants classified as having ADHD demonstrated marked difficulties in both academic and behavioral performance compared to their peers without ADHD symptoms. As presented in Figure 2 and Figure 3, the ADHD group was far more likely to be rated as “somewhat of a problem” or “problematic” across domains such as overall school performance, reading, writing, and mathematics, while the non-ADHD group was predominantly rated as “excellent” or “above average.” Similar patterns were observed in relationships with parents, siblings, and peers as well as participation in organized activities, highlighting that ADHD was associated with broader functional impairments beyond academics.

Table 3 reveals that socio-demographic variables such as age, sex, caregiver relationship, and caregiver education did not differ significantly across the inattentive, hyperactive/impulsive, and combined subtypes when compared with children and adolescents without ADHD symptoms. However, clinical and perinatal factors showed significant differences. For example, a history of meningitis was strongly associated with the inattentive and combined subtypes (*p* < 0.001 and *p* = 0.02, respectively), whereas abnormal brain imaging findings were significantly more common in the inattentive and hyperactive/impulsive groups (*p* < 0.001). Birth weight and mode of delivery also varied across groups; notably, children and adolescents with the combined subtype were more likely to have been born at very low birth weights through emergency cesarean sections (*p* = 0.04).

In addition, family psychiatric history emerged as a consistent risk factor across all ADHD subtypes compared to participants without ADHD symptoms. Nearly half of the participants with combined ADHD and 44.2% of those with the inattentive subtype reported a family history of psychiatric or neurological disorders, compared with only 16.9% in the non-ADHD group (*p* < 0.001). Head trauma was also significantly associated with the hyperactive/impulsive and inattentive subtypes, whereas prolonged screen time did not differ significantly across groups.

**Figure 4:**
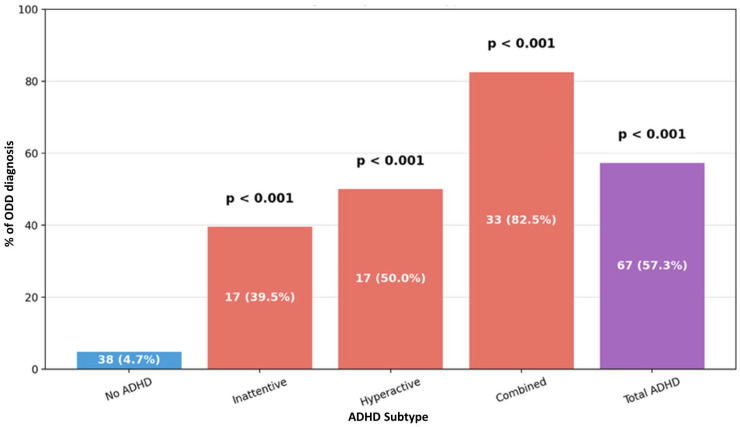
Distribution of ADHD subtypes in relation to oppositional defiant disorder (ODD).

**Figure 5:**
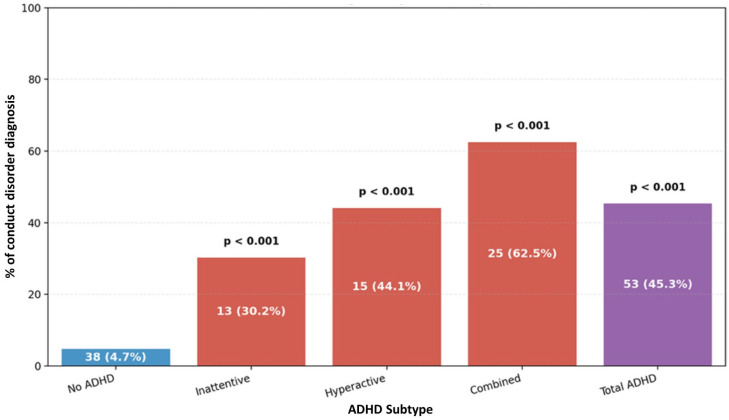
Distribution of ADHD subtypes in relation to conduct disorder.

**Figure 6:**
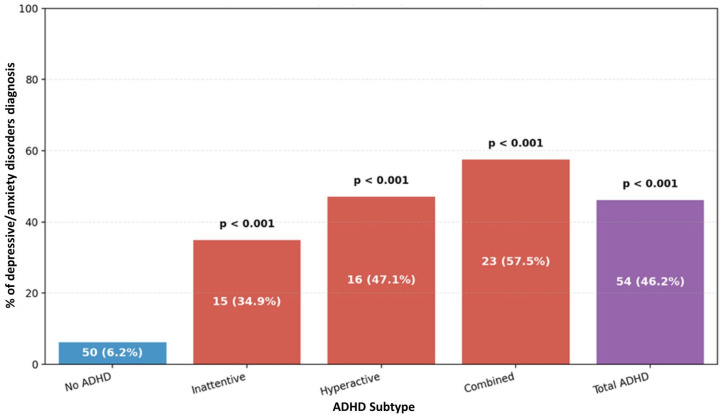
Distribution of ADHD subtypes in relation to depressive/anxiety disorders.

Comorbid psychiatric disorders were markedly more common among participants with ADHD symptoms than those without, with clear differences observed across ADHD subtypes. Overall, ODD was observed in 57.3% of participants in the ADHD group compared to 4.7% of the participants in the non-ADHD group (*p* < 0.001). When examined by subtype, more than four of five participants with the combined subtype (82.5%) had ODD, making it the most affected group, followed by the hyperactive/impulsive (50.0%) and inattentive (39.5%) subtypes, as presented in Figure 4. A similar trend was observed for conduct disorder, which affected 45.3% of the ADHD cases overall versus 4.7% in the non-ADHD group (*p* < 0.001). Once again, the combined subtype showed the highest burden (62.5%), followed by the hyperactive/impulsive subtype (44.1%), and the inattentive subtype (30.2%) as presented in Figure 5. Finally, depressive and anxiety disorders were disproportionately reported in participants with ADHD (46.2% vs. 6.2%, *p* < 0.001). Consistent with the previous pattern, the combined subtype showed the greatest vulnerability: 57.5% had a depressive/anxiety diagnosis, compared to 47.1% of participants with hyperactive/impulsive subtype and 34.9% of those with the inattentive subtype, as shown in Figure 6.

As shown in Table 4, the multivariate logistic regression analysis identified several independent predictors of ADHD after adjusting for potential confounders. Younger age was significantly associated with higher odds of ADHD (AOR = 0.925, 95% CI: 0.862–0.992, *p* = 0.029). The presence of previous psychiatric comorbidities remained one of the strongest predictors; children and adolescents with another psychiatric disorder had nearly a fourfold increase in ADHD risk (AOR = 3.951, 95% CI: 1.498–10.42, *p* = 0.005). Likewise, children with a prior ADHD diagnosis also had higher odds (AOR = 3.561, 95% CI: 2.009–6.311, *p* < 0.001). Low birth weight < 1.5 kg emerged as another significant factor (AOR = 3.294, 95% CI: 1.606–6.757, *p* = 0.0011), reinforcing the influence of early developmental risk. In contrast, delivery mode and previous meningitis were no longer significant in the adjusted model, suggesting that their effects may be mediated through other variables. A family history of mental, behavioral, or neurological disorders was also independently predictive, with affected participants showing more than twice the odds of developing ADHD when such a history was present (AOR = 2.72, 95% CI: 1.482–4.992, *p* = 0.001). Similarly, children and adolescents who engaged in 1–2 h of screen time per day also showed an elevated risk of ADHD compared to those who spent more than 3 h of screen time (AOR = 1.639, 95% CI: 1.015–2.645, *p* = 0.043).

## 4. Discussion

In a school-aged sample of 920 children and adolescents from Saudi Arabia’s Eastern Province, 12.7% met our case definition for probable ADHD on the Arabic Vanderbilt-based instrument, with the inattentive presentation being the most common. Similarly, recent national syntheses reported mid-to-high single-digit to low-double-digit ADHD prevalence in Saudi community samples [10]. Functionally, children with ADHD show marked academic and interpersonal impairments across multiple domains; this finding is consistent with prior literature demonstrating significant deficits in school achievement, task persistence, and classroom functioning in children with ADHD [11,12]. In the multivariate analysis, several factors remained independently associated with ADHD: younger age (AOR 0.93/year), very low birth weight <1.5 kg (AOR 3.29), any prior psychiatric diagnosis, whether a prior ADHD diagnosis (AOR 3.56) or other psychiatric disorders (AOR 3.95), and a positive family history of mental or neurologic disorders (AOR 2.72). The association with very low birth weight (VLBW) echoes developmental evidence identifying VLBW and very preterm birth as robust predictors of later ADHD symptoms [13]. Similarly, the clustering of ADHD with prior psychiatric diagnoses and family psychiatric vulnerability aligns with genetic and familial aggregation studies that have demonstrated high heritability and cross-diagnostic co-occurrence [14,15]. Conversely, signals observed in the bivariate analyses for emergency cesarean delivery, head trauma, meningitis, and abnormal neuroimaging did not persist after adjustment. This divergence, relative to the more stable perinatal and familial factors, suggests that these associations may have been confounded by underlying developmental risk. In addition, a small but statistically significant increase in odds was observed for 1–2 h per day of screen time compared with >3 h per day. Although this pattern is counterintuitive, it warrants cautious interpretation; recent regional work indicates heterogeneous behavioral effects of screen exposure depending on duration, content, and parental mediation [16,17]. Overall, this pattern supports ADHD as a neurodevelopmental condition clustered within familial/psychiatric vulnerability and perinatal risk, with broad functional sequelae affecting school performance and peer/family relationships.

The contribution of this study should be interpreted primarily as contextual and epidemiologic rather than as the identification of entirely new ADHD predictors. Several of the factors that remained associated with probable ADHD in our adjusted model, including very low birth weight, prior psychiatric history, and family psychiatric or neurologic vulnerability, are broadly consistent with international evidence. However, data from the Eastern Province of Saudi Arabia remain limited compared with other settings, and prevalence estimates can vary substantially according to age range, sampling frame, informant, and assessment instrument. Our findings therefore extend the international literature by showing how ADHD prevalence, subtype distribution, and associated clinical burden present in an understudied Middle Eastern regional population assessed with a validated Arabic Vanderbilt-based approach across a broad developmental age range.

Our findings are consistent with a large meta-analysis of ADHD in Saudi Arabia (n = 455,334), which reported an overall prevalence of 12.4%, with inattentive subtypes being higher than hyperactivity subtypes (2.9% vs. 2.5%) [18]. Compared with our estimate (12.7% ADHD, with the inattentive subtype being the most common), Jenahi’s all-girls sample (*n* = 1009) from Al-Khobar reported a much lower overall prevalence (3.5%), with subtype proportions that differed by domain and age. That study also reported decreasing ADHD rates with increasing age and a higher burden in larger families and later birth orders [8]. Our higher estimates are likely driven mainly by the inclusion of a mixed population of boys and girls across a wider geographical area, consistent with evidence that boys are at three or more times higher risk of ADHD than girls [19]. Similarly, a large nationwide study from the USA (60,358 children) supported our findings on the association between low birth weight and ADHD. That study reported that low birth weight children had significantly higher odds of ADHD compared to those with normal birth weight [aOR = 1.32 (95% CI, 1.03–1.68)] while smaller-sample studies have reported odds as high as 3.8 times greater than normal weight peers [20,21].

AlZaben et al. used the teacher-completed Vanderbilt (VADTRS) across randomly selected public schools in Jeddah (*n* = 929) [6]. They reported an overall ADHD prevalence of 5.0%, with the combined subtype most common at 2.7% (hyperactive-impulsive 1.2%, inattentive 1.1%). They also noted high comorbidity burdens, including ODD/CD at 56.5%, impaired academic performance at 54.4%, classroom behavioral problems at 44.4%, and anxiety/depression at 41.3%. In contrast, our Eastern Province survey yielded a higher point prevalence of 12.7%, and a different subtype pattern, with the inattentive presentation ranking first, followed by the combined and then hyperactive/impulsive subtypes. Comorbidities in our cohort were similar to or higher than those reported by AlZaben et al. for both externalizing and internalizing symptoms: ODD occurred in 57.3% overall (82.5% combined, 50.0% hyperactive/impulsive, and 39.5% inattentive) and anxiety/depression occurred in 46.2% overall (57.5% combined, 47.1% hyperactive/impulsive, and 34.9% inattentive), closely mirroring or exceeding AlZaben’s teacher-rated Vanderbilt estimates. We relied on caregiver reports (using the Arabic Vanderbilt/SNAP-IV structured) and incorporated impairment and cross-setting criteria. We also captured prior diagnoses, medication use, neurodevelopmental history, and perinatal exposures using regression modeling. Studies report that behavioral characteristics differ between home and school; therefore, differences in methodologies can explain these differences in prevalence and subtypes, combined with the differences between Jeddah and the Eastern Province [22,23,24].

AlShamrani et al. screened 1430 children aged 6–10 years in the Eastern Province and identified 185 as having ADHD (12.9%), a rate similar to our estimates. They reported a higher prevalence in males and documented associations with family history, maternal/second-hand smoking, preterm birth, neurologic illness, and trauma-related risk gradients, including strong family history signals, head injury, neuroimaging abnormalities, and functional impairment across school and peer relationships [7]. Although both studies were cross-sectional and conducted in the same region, important methodological differences exist. Specifically, AlShamrani et al. used an online parent survey based on DSM-5 criteria and the Conners scale, excluded those with known developmental/mental disorders at the onset, and focused on screening positivity and risk factors. By contrast, our approach employed a structured Vanderbilt/SNAP-IV framework, incorporating an explicit impairment criterion (≥1 performance item rated ≥ 4) and a requirement for symptom presence across “multiple settings”, and collected more detailed perinatal and neurologic history. We also used multivariable regression, which provided clearer and more robust inferences than the simple test of significance performed by AlShamrani et al. Collectively, these design elements (especially the inclusion of impairment and cross-setting criteria) make our estimates more conservative and accurate.

Furthermore, our findings are consistent with those of previous Saudi studies (based on the meta-analysis by Aljadani et al.), which found no significant effect of sex on ADHD diagnosis when using validated screening tools. This challenges the widely held assumption that females are inherently less prone to ADHD than males [25]. Although prior studies have shown that a higher ratio of males to females with ADHD diagnoses (ranging from 2:1 to 9:1) is common in childhood populations and clinical studies, these disparities diminish in adulthood, and many females who meet screening cutoff scores are never clinically diagnosed [19,26,27]. This pattern is consistent with evidence that girls more often present with quieter, less disruptive inattentive symptoms; meet impairment in only one setting (home or school) rather than both; and have attention-related difficulties “overshadowed” by co-occurring anxiety or mood symptoms. Consequently, when broad screening is applied and DSM-5 criteria are assessed systematically across informants, sex-based diagnostic biases tend to shrink.

The international relevance of our findings also lies in the broader clinical profile captured in this study. Beyond estimating prevalence, we found that probable ADHD in this sample was associated with substantial academic and interpersonal impairment and with high levels of comorbid oppositional, conduct, and anxiety/depressive symptoms. In addition, the inattentive presentation was the most common subtype in our cohort, whereas prior Saudi studies using different informants and sampling strategies have reported different subtype patterns and lower overall prevalence estimates. Taken together, these findings underscore the importance of generating data from diverse cultural and methodological contexts, because they help refine cross-national comparisons and strengthen the external validity of the ADHD literature by showing both the consistency of core associations and the variability of epidemiologic expression across settings.

### 4.1. Strengths and Limitations

Our study adds regionally relevant evidence by sampling a large mixed-sex community cohort from the Eastern Province. The study also leveraged multivariate logistic regression with a comprehensive set of perinatal, neurological, and family variables to clarify which associations remained independent. However, several limitations must be acknowledged when interpreting the findings of this study. The cross-sectional design precludes any determination of temporal or causal relationships. Moreover, the online, caregiver-reported methodology renders the study vulnerable to selection bias, recall bias, and common-rater bias, which may have influenced both exposure and outcome reporting. The absence of clinician-administered diagnostic interviews further limits diagnostic accuracy and may have resulted in misclassification of ADHD status. Although multiple covariates were included in the analyses, residual confounding remains possible. Relevant factors such as socioeconomic adversity and unrecognized learning disorders may not have been comprehensively captured. In addition, certain exposures (e.g., lead exposure or neurological illness) were based solely on proxy reports without biomarker confirmation or medical record verification, increasing the potential for measurement error and residual confounding. Small sample sizes within specific ADHD subtypes reduced statistical power for subgroup analyses, limiting the robustness of some comparisons. Furthermore, the use of multiple hypothesis testing increases the risk of type I error and the identification of spurious associations, including potentially counterintuitive findings such as those related to screen time. The generalizability of the findings may also be restricted to children and adolescents in the Eastern Province and may not extend to non-enrolled youth or populations in other regions. Finally, the study did not incorporate structured assessments of academic or functional performance, nor did it include objective cognitive testing. As a result, the alignment between reported symptom severity and actual functional impairment could not be directly evaluated.

### 4.2. Implications and Future Directions

Clinically, the high symptom burden and clear functional impairment observed in our cohort suggest the need for earlier school-based identification pathways and enhanced caregiver education, along with routine screening for oppositionality, conduct problems, and internalizing symptoms, particularly in combined-type presentations. Perinatal history (especially very low birth weight) and family psychiatric history should be integrated into risk stratification and anticipatory guidance. At the system level, the findings support coordinated pediatric school mental health services and referral pathways that do not rely solely on teacher-initiated concerns. Looking ahead, future research should employ longitudinal designs with clinician-confirmed diagnoses to establish incidence, persistence, and causal pathways. Further studies should also incorporate objective cognitive/behavioral tasks and examine potentially modifiable environmental factors such as sleep patterns, screen content and timing, and classroom support.

## 5. Conclusions

In this large community sample, ADHD symptoms, predominantly inattentive, were common and functionally consequential and were independently associated with developmental vulnerability (younger age, very low birth weight), family psychiatric loading, and concurrent psychiatric morbidity. This study extends the international ADHD literature by contributing contemporary data from an underrepresented Middle Eastern setting and by showing that, in this population, probable ADHD is common, functionally impairing, and clustered with recognizable developmental, psychiatric, and familial vulnerabilities. Overall, the profile underscores the need for early detection, holistic assessment of comorbidities, and school-anchored interventions to mitigate the educational and social impacts.

## Figures and Tables

**Table 1:** Comparative analysis of socio-demographic characteristics of study population with ADHD symptoms vs. those with no ADHD symptoms.

Study Variables	Total Sample	ADHD		Test of Sig.	*p*-Value
		Yes (*n* = 117)	No (*n* = 803)		
Age of the child	10.71 ± 3.96	10.64 ± 3.9	10.72 ± 3.97	0.347	0.556
Sex of the child				0.5	0.48
Male	487 (52.9%)	66 (56.4%)	421 (52.4%)		
Female	433 (47.1%)	51 (43.6%)	382 (47.6%)		
Relationship with the patient				3.826	0.148
Mother	516 (56.1%)	71 (60.7%)	445 (55.4%)		
Father	152 (16.5%)	12 (10.3%)	140 (17.4%)		
Other relative	252 (27.4%)	34 (29.1%)	218 (27.1%)		
Age of the relative				2.599	0.761
Older than 40 years	269 (29.2%)	37 (31.6%)	232 (28.9%)		
36–40 years	176 (19.1%)	26 (22.2%)	150 (18.7%)		
31–35 years	177 (19.2%)	18 (15.4%)	159 (19.8%)		
26–30 years	133 (14.5%)	14 (12.0%)	119 (14.8%)		
20–25 years	142 (15.4%)	19 (16.2%)	123 (15.3%)		
Less than 20 years	23 (2.5%)	3 (2.6%)	20 (2.5%)		
Level of education				2.29	0.808
PhD	63 (6.9%)	10 (8.5%)	53 (6.6%)		
Master’s degree	95 (10.3%)	12 (10.3%)	83 (10.3%)		
Bachelor’s degree	510 (55.5%)	63 (53.8%)	447 (55.7%)		
Diploma	110 (12.0%)	13 (11.1%)	97 (12.1%)		
High school	134 (14.6%)	17 (14.5%)	117 (14.6%)		
Illiterate	7 (0.8%)	2 (1.7%)	5 (0.6%)		
Known ADHD or any other psychiatric disorder				73.868	**<0.001**
No	683 (74.2%)	49 (41.9%)	634 (79.0%)		
ADHD	193 (21.0%)	54 (46.2%)	139 (17.3%)		
Other psychiatric disorders	44 (4.8%)	14 (12.0%)	30 (3.7%)		

Bold values indicate statistically significant results (*p* < 0.05).

**Table 2:** Comparative analysis of other potential risk factors of study population with ADHD symptoms vs. those with no ADHD symptoms.

Study Variables	Total Sample	ADHD		Test of Sig.	*p*-Value
		Yes (*n* = 117)	No (*n* = 803)		
What was the child’s weight at birth?				8.328	0.08
More than 4 kg	56 (6.1%)	7 (6.0%)	49 (6.1%)		
Between 4 kg and 2.5 kg	428 (46.5%)	59 (50.4%)	369 (46.0%)		
Between 2.5 kg and 1.5 kg	238 (25.9%)	22 (18.8%)	216 (26.9%)		
Less than 1.5 kg	46 (5.0%)	11 (9.4%)	35 (4.4%)		
Unknown	152 (16.5%)	18 (15.4%)	134 (16.7%)		
Mode of delivery				8.763	**0.033**
Normal vaginal delivery	568 (61.7%)	67 (57.3%)	501 (62.4%)		
Elective cesarean section	183 (19.9%)	19 (16.2%)	164 (20.4%)		
Emergency C/section	107 (11.6%)	23 (19.7%)	84 (10.5%)		
Unknown	62 (6.7%)	8 (6.8%)	54 (6.7%)		
At what gestational age was this childborn (Preterm/term)?				2.765	0.251
Born at or after 37 weeks of gestation	564 (61.3%)	72 (61.5%)	492 (61.3%)		
Born before 37 weeks of gestation	134 (14.6%)	22 (18.8%)	112 (13.9%)		
Unknown	222 (24.1%)	23 (19.7%)	199 (24.8%)		
Does the child/adolescent have a previous brain infection “meningitis”?				10.241	**0.006**
Yes	49 (5.3%)	8 (6.8%)	41 (5.1%)		
No	790 (85.9%)	90 (76.9%)	700 (87.2%)		
Unknown	81 (8.8%)	19 (16.2%)	62 (7.7%)		
Does the child/adolescent have a history of head trauma?				13.069	**0.0015**
Yes	86 (9.3%)	15 (12.8%)	71 (8.8%)		
No	738 (80.2%)	80 (68.4%)	658 (81.9%)		
Unknown	96 (10.4%)	22 (18.8%)	74 (9.2%)		
Does the child/adolescent have a previous abnormal brain imaging (CT, MRI, cranial US)?				16.577	**<0.001**
Yes	62 (6.7%)	11 (9.4%)	51 (6.4%)		
No	772 (83.9%)	84 (71.8%)	688 (85.7%)		
Unknown	86 (9.3%)	22 (18.8%)	64 (8.0%)		
Did the mother have any exposure to Tobacco, Alcohol, or recreational medications during her pregnancy?				4.627	0.099
Yes	46 (5.0%)	9 (7.7%)	37 (4.6%)		
No	797 (86.6%)	94 (80.3%)	703 (87.5%)		
Unknown	77 (8.4%)	14 (12.0%)	63 (7.8%)		
Is there a family history (from maternal/paternal side) of any mental, behavioral, neurological disorders?				56.935	**<0.001**
Yes	184 (20.0%)	48 (41.0%)	136 (16.9%)		
No	617 (67.1%)	43 (36.8%)	574 (71.5%)		
Unknown	119 (12.9%)	26 (22.2%)	93 (11.6%)		
How many hours does the child/adolescent spend on screens daily (TV, smartphones, tablets)?				5.835	0.212
More than 3 h	417 (45.3%)	65 (55.6%)	352 (43.8%)		
Between 2–3 h	213 (23.2%)	23 (19.7%)	190 (23.7%)		
Between 1–2 h	171 (18.6%)	18 (15.4%)	153 (19.1%)		
Less than 1 h	76 (8.3%)	7 (6.0%)	69 (8.6%)		
None	43 (4.7%)	4 (3.4%)	39 (4.9%)		

Bold values indicate statistically significant results (*p* < 0.05).

**Table 3:** Comparative analysis of socio-demographic characteristics and risk factors of ADHD subtypes.

Study Variables	No ADHD (*n* = 803)	Inattention (*n* = 43)	*p*-Value	Hyperactivity (*n* = 34)	*p*-Value	Combined (*n* = 40)	*p*-Value
Age of the child	10.72 ± 3.97	11.26 ± 3.72	0.363	10.65 ± 4.07	0.919	9.98 ± 3.9	0.249
Sex of the child			0.936		0.411		0.513
Male	421 (52.4%)	26 (60.5%)		18 (52.9%)		22 (55.0%)	
Female	382 (47.6%)	17 (39.5%)		16 (47.1%)		18 (45.0%)	
Relation to patient			0.113		0.31		0.24
Mother	445 (55.4%)	27 (62.8%)		23 (67.6%)		21 (52.5%)	
Father	140 (17.4%)	6 (14.0%)		3 (8.8%)		3 (7.5%)	
Other relative	218 (27.1%)	10 (23.3%)		8 (23.5%)		16 (40.0%)	
Age of the relative			0.713		0.239		0.834
Older than 40 years	232 (28.9%)	13 (30.2%)		7 (20.6%)		17 (42.5%)	
36–40 years	150 (18.7%)	13 (30.2%)		6 (17.6%)		7 (17.5%)	
31–35 years	159 (19.8%)	6 (14.0%)		9 (26.5%)		3 (7.5%)	
26–30 years	119 (14.8%)	6 (14.0%)		3 (8.8%)		5 (12.5%)	
20–25 years	123 (15.3%)	5 (11.6%)		7 (20.6%)		7 (17.5%)	
Less than 20 years	20 (2.5%)	0 (0.0%)		2 (5.9%)		1 (2.5%)	
Level of education			0.344		0.975		0.247
PhD	53 (6.6%)	7 (16.3%)		3 (8.8%)		0 (0.0%)	
Master’s degree	83 (10.3%)	6 (14.0%)		4 (11.8%)		2 (5.0%)	
Bachelor’s degree	447 (55.7%)	20 (46.5%)		16 (47.1%)		27 (67.5%)	
Diploma	97 (12.1%)	5 (11.6%)		4 (11.8%)		4 (10.0%)	
High school	117 (14.6%)	5 (11.6%)		6 (17.6%)		6 (15.0%)	
Illiterate	5 (0.6%)	0 (0.0%)		1 (2.9%)		1 (2.5%)	
Known ADHD or any other psychiatric disorder			**<0.001**		**<0.001**		**<0.001**
No	634 (79.0%)	14 (32.6%)		16 (47.1%)		19 (47.5%)	
ADHD	139 (17.3%)	25 (58.1%)		12 (35.3%)		17 (42.5%)	
Other psychiatric disorders	30 (3.7%)	4 (9.3%)		6 (17.6%)		4 (10.0%)	
What was the child’s weight at birth?			0.063		0.7		**0.04**
More than 4 kg	49 (6.1%)	2 (4.7%)		1 (2.9%)		4 (10.0%)	
Between 4 kg and 2.5 kg	369 (46.0%)	21 (48.8%)		17 (50.0%)		21 (52.5%)	
Between 2.5 kg and 1.5 kg	216 (26.9%)	10 (23.3%)		6 (17.6%)		6 (15.0%)	
Less than 1.5 kg	35 (4.4%)	3 (7.0%)		7 (20.6%)		1 (2.5%)	
Unknown	134 (16.7%)	7 (16.3%)		3 (8.8%)		8 (20.0%)	
Mode of delivery			**0.01**		0.735		**0.04**
Normal vaginal delivery	501 (62.4%)	24 (55.8%)		18 (52.9%)		25 (62.5%)	
Elective cesarean section	164 (20.4%)	10 (23.3%)		4 (11.8%)		5 (12.5%)	
Emergency cesarean section	84 (10.5%)	6 (14.0%)		11 (32.4%)		6 (15.0%)	
Unknown	54 (6.7%)	3 (7.0%)		1 (2.9%)		4 (10.0%)	
At what gestational age was this child born (Preterm/term)?			0.343		0.958		**0.03**
Born at or after 37 weeks of gestation	492 (61.3%)	29 (67.4%)		20 (58.8%)		23 (57.5%)	
Born before 37 weeks of gestation	112 (13.9%)	7 (16.3%)		10 (29.4%)		5 (12.5%)	
Unknown	199 (24.8%)	7 (16.3%)		4 (11.8%)		12 (30.0%)	
Does the child/adolescent have a previous brain infection, “meningitis”?			**<0.001**		0.512		**0.02**
Yes	41 (5.1%)	2 (4.7%)		3 (8.8%)		3 (7.5%)	
No	700 (87.2%)	37 (86.0%)		22 (64.7%)		31 (77.5%)	
Unknown	62 (7.7%)	4 (9.3%)		9 (26.5%)		6 (15.0%)	
Does the child/adolescent have a history of head trauma?			**<0.001**		**0.04**		0.133
Yes	71 (8.8%)	3 (7.0%)		5 (14.7%)		7 (17.5%)	
No	658 (81.9%)	34 (79.1%)		22 (64.7%)		24 (60.0%)	
Unknown	74 (9.2%)	6 (14.0%)		7 (20.6%)		9 (22.5%)	
Does the child/adolescent have a previous abnormal brain imaging (CT, MRI, cranial US)?			**<0.001**		**<0.001**		0.124
Yes	51 (6.4%)	3 (7.0%)		3 (8.8%)		5 (12.5%)	
No	688 (85.7%)	35 (81.4%)		24 (70.6%)		25 (62.5%)	
Unknown	64 (8.0%)	5 (11.6%)		7 (20.6%)		10 (25.0%)	
Did the mother have any exposure to Tobacco, Alcohol, or recreational medications during her pregnancy?			0.175		0.125		0.378
Yes	37 (4.6%)	4 (9.3%)		2 (5.9%)		3 (7.5%)	
No	703 (87.5%)	35 (81.4%)		28 (82.4%)		31 (77.5%)	
Unknown	63 (7.8%)	4 (9.3%)		4 (11.8%)		6 (15.0%)	
Is there a family history (from maternal/paternal side) of any mental, behavioral, neurological disorders?			**<0.001**		**<0.001**		**<0.001**
Yes	136 (16.9%)	19 (44.2%)		11 (32.4%)		18 (45.0%)	
No	574 (71.5%)	18 (41.9%)		12 (35.3%)		13 (32.5%)	
Unknown	93 (11.6%)	6 (14.0%)		11 (32.4%)		9 (22.5%)	
How many hours does the child/adolescent spend on screens daily (TV, smartphones, tablets)?			0.354		0.078		0.873
More than 3 h	352 (43.8%)	23 (53.5%)		15 (44.1%)		27 (67.5%)	
Between 2–3 h	190 (23.7%)	9 (20.9%)		9 (26.5%)		5 (12.5%)	
Between 1–2 h	153 (19.1%)	7 (16.3%)		6 (17.6%)		5 (12.5%)	
Less than 1 h	69 (8.6%)	3 (7.0%)		3 (8.8%)		1 (2.5%)	
None	39 (4.9%)	1 (2.3%)		1 (2.9%)		2 (5.0%)	

Bold values indicate statistically significant results (*p* < 0.05).

**Table 4:** Multivariable logistic regression analysis of predictive factors of participants with ADHD.

Factor	Unadjusted OR (95% CI)	*p*-Value	Adjusted OR (95% CI)	*p*-Value
Age of the child	0.995 (0.947–1.045)	0.841	0.925 (0.862–0.992)	**0.029**
Female	0.852 (0.576–1.259)	0.42	0.737 (0.429–1.265)	0.269
Relationship with the patient (reference = Mother)				
Father	0.537 (0.283–1.019)	0.057	0.521 (0.217–1.255)	0.146
Other relative	0.978 (0.63–1.517)	0.919	1.039 (0.55–1.965)	0.906
Level of education (reference = Bachelor’s degree)				
PhD	1.339 (0.648–2.765)	0.431	1.749 (0.695–4.399)	0.235
Master’s degree	1.026 (0.53–1.985)	0.94	0.707 (0.281–1.779)	0.461
Diploma	0.951 (0.503–1.796)	0.877	0.637 (0.264–1.536)	0.315
High school	1.031 (0.581–1.829)	0.917	0.859 (0.387–1.905)	0.709
Illiterate	2.838 (0.539–14.94)	0.218	0.671 (0.081–5.593)	0.713
Known psychiatric disorder (reference = No)				
ADHD	5.027 (3.276–7.712)	**<0.001**	3.561 (2.009–6.311)	**<0.001**
Other psychiatric disorders	6.038 (3.005–12.132)	**<0.001**	3.951 (1.498–10.42)	**0.005**
What was the child’s weight at birth? (reference = Between 4 kg and 2.5 kg)				
More than 4 kg	1.411 (0.735, 2.704)	0.3	1.272 (0.615–2.629)	0.515
Between 2.5 kg and 1.5 kg	1.013 (0.678, 1.515)	0.947	0.986 (0.636–1.531)	0.953
Less than 1.5 kg	5.037 (2.688, 9.441)	**<0.001**	3.294 (1.606–6.757)	**0.0011**
Unknown	0.873 (0.537, 1.419)	0.585	0.669 (0.354–1.265)	0.217
Mode of delivery (reference = Normal vaginal)				
Elective cesarean section	0.866 (0.505–1.485)	0.602	0.544 (0.25–1.185)	0.125
Emergency cesarean section	2.047 (1.209–3.468)	**0.008**	1.916 (0.907–4.05)	0.089
Unknown	1.108 (0.505–2.429)	0.798	0.712 (0.181–2.805)	0.628
Gestational age (reference = Born at or after 37 weeks of gestation)				
Born before 37 weeks of gestation	1.342 (0.798–2.257)	0.267	0.64 (0.296–1.384)	0.257
Unknown	0.79 (0.48–1.299)	0.352	0.985 (0.462–2.102)	0.969
Previous meningitis (reference = No)				
Unknown	2.384 (1.363–4.168)	**0.002**	1.342 (0.461–3.909)	0.59
Yes	1.518 (0.69–3.34)	0.3	0.517 (0.146–1.833)	0.307
History of head trauma (reference = No)				
Unknown	2.445 (1.44–4.152)	**<0.001**	1.128 (0.405–3.145)	0.817
Yes	1.738 (0.95–3.177)	0.073	1.273 (0.49–3.307)	0.62
Abnormal brain imaging (reference = No)				
Unknown	2.815 (1.649–4.806)	**<0.001**	1.302 (0.419–4.045)	0.648
Yes	1.767 (0.886–3.521)	0.106	0.385 (0.119–1.242)	0.11
Maternal exposure during pregnancy (reference = No)				
Unknown	1.662 (0.896–3.083)	0.107	0.321 (0.098–1.054)	0.061
Yes	1.819 (0.851–3.888)	0.123	1.003 (0.287–3.505)	0.996
Family history of disorders (reference = No)				
Unknown	3.732 (2.188–6.365)	**<0.001**	1.915 (0.807–4.542)	0.14
Yes	4.711 (2.998–7.404)	**<0.001**	2.72 (1.482–4.992)	**0.001**
Screen time (reference = More than 3 h)				
Between 2–3 h	0.993 (0.642–1.535)	0.977	1.061 (0.662–1.701)	0.805
Between 1–2 h	1.739 (1.141–2.652)	**0.01**	1.639 (1.015–2.645)	**0.043**
Less than 1 h	2.756 (1.622–4.684)	0.051	2.356 (1.271–4.367)	0.6
None	1.251 (0.575–2.721)	0.572	0.68 (0.278–1.662)	0.398

Bold values indicate statistically significant results (*p* < 0.05).

## Data Availability

The original contributions presented in this study are included in this article. Further inquiries can be directed to the corresponding authors.
